# A comparison of 12-gene colon cancer assay gene expression in African American and Caucasian patients with stage II colon cancer

**DOI:** 10.1186/s12885-016-2365-3

**Published:** 2016-06-18

**Authors:** Rangaswamy Govindarajan, James Posey, Calvin Y. Chao, Ruixiao Lu, Trafina Jadhav, Ahmed Y. Javed, Awais Javed, Fade A. Mahmoud, Raymond U. Osarogiagbon, Upender Manne

**Affiliations:** University of Arkansas for Medical Sciences, 4301 W Markham, Slot 508, Little Rock, AR 72205 USA; University of Alabama at Birmingham Comprehensive Cancer Center, Birmingham, AL USA; Genomic Health Inc., Redwood City, CA; Genomic Health, Inc., Redwood City, CA USA; Boston Baskin Cancer Foundation, Baptist Cancer Center, Memphis, TN USA

**Keywords:** Colon cancer, 12-gene assay, Gene expression, Stage II, African-American, Caucasian

## Abstract

**Background:**

African American (AA) colon cancer patients have a worse prognosis than Caucasian (CA) colon cancer patients, however, reasons for this disparity are not well understood. To determine if tumor biology might contribute to differential prognosis, we measured recurrence risk and gene expression using the Onco*type* DX*®* Colon Cancer Assay (12-gene assay) and compared the Recurrence Score results and gene expression profiles between AA patients and CA patients with stage II colon cancer.

**Methods:**

We retrieved demographic, clinical, and archived tumor tissues from stage II colon cancer patients at four institutions. The 12-gene assay and mismatch repair (MMR) status were performed by Genomic Health (Redwood City, California). Student’s *t*-test and the Wilcoxon rank sum test were used to compare Recurrence Score data and gene expression data from AA and CA patients (SAS Enterprise Guide 5.1).

**Results:**

Samples from 122 AA and 122 CA patients were analyzed. There were 118 women (63 AA, 55 CA) and 126 men (59 AA, 67 CA). Median age was 66 years for AA patients and 68 for CA patients. Age, gender, year of surgery, pathologic T-stage, tumor location, the number of lymph nodes examined, lymphovascular invasion, and MMR status were not significantly different between groups (*p* = 0.93). The mean Recurrence Score result for AA patients (27.9 ± 12.8) and CA patients (28.1 ± 11.8) was not significantly different and the proportions of patients with high Recurrence Score values (≥41) were similar between the groups (17/122 AA; 15/122 CA). None of the gene expression variables, either single genes or gene groups (cell cycle group, stromal group, BGN1, FAP, INHBA1, Ki67, MYBL2, cMYC and GADD45B), was significantly different between the racial groups. After controlling for clinical and pathologic covariates, the means and distributions of Recurrence Score results and gene expression profiles showed no statistically significant difference between patient groups.

**Conclusion:**

The distribution of Recurrence Score results and gene expression data was similar in a cohort of AA and CA patients with stage II colon cancer and similar clinical characteristics, suggesting that tumor biology, as represented by the 12-gene assay, did not differ between patient groups.

## At a glance

- Tumor biology as reflected in differential gene expression may contribute to differential outcomes for African-American patients as compared to Caucasian colon cancer patients.
- A cohort of patients well balanced for clinical and demographic factors was selected for gene expression testing using the Onco*type* DX colon cancer assay as a measure of tumor biology.
- The distribution of Recurrence Score results for African-American patients (*n* = 122) was not significantly different than that of Caucasian patients (*n* = 122).
- Expression of single genes or gene groups (cell cycle group, stromal group, BGN1, FAP, INHBA1, Ki67, MYBL2, cMYC and GADD45B) also did not differ significantly between ethnic groups.
- Although differences in outcomes have been observed between AA and CA patients, this study found no difference in tumor biology as represented by the 12-gene assay when no differences in demographic or clinical factors were present.

## Background

Racial disparities in the outcomes of many cancers are a well-recognized phenomenon. Although the survival of patients with colorectal cancer has improved in recent years, disparity in outcomes between African American (AA) and Caucasian (CA) patients persists [1]. This may be due to a variety of factors, such as socioeconomic factors influencing access to good quality care, differences in screening participation, or tumor biology [2–5].

The Onco*type* DX® Colon Cancer Assay (12-gene assay, Genomic Health, Inc., Redwood City, California) is a 12-gene RT-PCR based test that yields a Recurrence Score® result that has been clinically validated to predict the probability of recurrence following resection of stage II and stage III colon cancer [6–8]. The 12 genes measured consist of 7 cancer-related genes, which include 3 cell cycle genes (MK167, MYBL2, and MYC), 3 stromal genes (BGN, INHBA and FAP), and an early response gene (GADD45B), and 5 reference normalization genes. The Recurrence Score result (ranging from 0 to 100 with lower values representing lower risk of recurrence) is derived from RNA expression levels of these genes as determined by RT-PCR in formalin-fixed paraffin-embedded (FFPE) tumor tissue using a quantitative algorithm. The assay has been shown to add significant information beyond conventional clinical and pathologic factors regarding the risk of recurrence and has been shown to be clinically valid in multiple studies [6–8].

The distribution of Recurrence Score® results and associated gene expression profiles based on race/ethnicity have not been previously assessed. We used the 12-gene assay as a measure of gene expression activity to evaluate possible biological differences between AA and CA patients with resected stage II colon cancer.

## Methods

Patients with resected stage II colon cancer and with archived tumor tissue were identified from tumor registries at four institutions (University of Arkansas for Medical Sciences, Little Rock, AR; Central Arkansas Veterans Healthcare System, Little Rock, AR; University of Tennessee Cancer Institute, Memphis, TN; and the University of Alabama at Birmingham, Birmingham, AL). Institutional Review Board approval was obtained from the respective institutions. Demographic and clinical data, including pathologic stage, was obtained by manual chart review. Two hundred ninety three stage II colon cancer patients, matched for the year of diagnosis, age, and sex, were selected for the study. Race/ethnicity was self-reported. Patients with rectal cancer and those with synchronous tumors were excluded from the study. Paraffin blocks or unstained sections on slides were obtained for the selected patients. After verification of the diagnosis and stage by an independent pathologist, the 12-gene assay and mismatch repair status (MMR) by immunohistochemistry for MLH1 and MSH2 were performed on these samples at the Genomic Health laboratory.

## Statistical methods

### Primary analysis

To address the primary objective of the study, the distributions of the Recurrence Score results for AA and CA patients were compared. Specifically, t-tests for two independent samples were used to determine if there were significant differences between the Recurrence Score results and the expression of individual genes in the two patient groups. If the normality assumption of the distributions of the Recurrence Score results was found to be invalid, a nonparametric Wilcoxon rank-sum test was used. In addition, the Recurrence Score distributions by race were summarized using histograms and descriptive statistics, such as means, medians, standard deviations, and ranges. Similar analyses were carried out to compare the expression levels of gene groups and individual genes within the 12-gene assay between the two patient groups.

### Secondary analyses

We compared the distribution of demographic and pathology variables between AA and CA patients using Chi-square tests for categorical variables and two-sided t-tests for continuous variables. We also compared the distributions of the Recurrence Score results, gene groups and individual genes between the two patient groups, controlling for demographic and pathologic characteristics. We used multiple linear regression models to evaluate the relationships of the continuous Recurrence Score value, gene groups, and individual genes to relevant demographic and pathologic covariates, including race, gender, age at surgery, number of nodes examined, pathologic T stage, MMR status, and lymphatic vascular invasion (LVI).

All tests of hypotheses were conducted at a two-sided alpha level of 0.05 unless otherwise noted. In this exploratory study, we have made no adjustments for multiple comparisons. All analyses were conducted with SAS 9.3 (SAS Institute, Cary, NC).

## Results

**Fig. 1:**
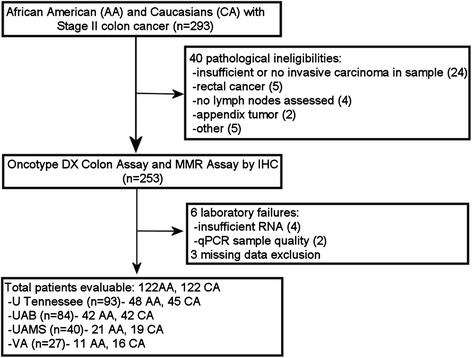
CONSORT diagram for the study. Study cohort of subjects selected for the study after exclusion of those who did not meet the selection criteria. The analysis was based on 244 samples (122 Caucasian patients and 122 African-American patients). IHC: immunohistochemistry; U Tennessee: University of Tennessee; UAB: University of Alabama Birmingham; UAMS: University of Arkansas for Medical Sciences; VA: Central Arkansas Veterans Healthcare System

Stage II colon cancer patients (*n* = 293) were selected from tumor registries of four institutions (Fig. 1). Forty patients were excluded from analysis at pathology review (24 with insufficient or no invasive cancer, 5 with rectal cancer, 4 with no lymph node data, 2 with appendicular cancers and 5 for other reasons). Six patients were excluded due to laboratory failures (4 for insufficient RNA and 2 for poor quantitative PCR quality), and 3 patients were excluded due to missing MMR testing results. The remaining 244 samples (from 122 AA and 122 CA patients) were used in the analysis. The racial distribution of patients from each institution is listed in Fig. 1.

In the cohort of 244 patients, there were 118 women (63 AA, 55 CA) and 126 men (59 AA, 67 CA) (Table 1). The median age at surgery was 66 years (range 35–88 years) for AA patients, and 68 years (range 40–97 years) for CA patients. Surgery was performed before the year 2000 in 31 % of AA and CA patients, from 2000 to 2009 in 40 % of AA and 49 % of CA patients, and from 2010 onwards in 29 % of AA and 20 % of CA patients. Table 1 shows the distribution of the demographic, clinical, and pathological characteristics of the patients eligible for analysis. Age, gender, year of surgery, pathologic stage, tumor location, number of nodes examined, LVI, and MMR status were not significantly different between the two racial groups as determined by Chi-square tests.

**Table 1:** Demographic and clinical characteristics

Characteristics	African American (AA)	Caucasian (CA)
Age at Surgery (Yrs.)		
Median (Range)	66 (35, 88)	68 (40, 97)
IQR (1^st^ quartile, 3^rd^ quartile)	21 (55–76)	20 (57–77)
Gender		
Female (*n* = 118)	63 (51.6 %)	55 (45.1 %)
Male (*n* = 126)	59 (48.4 %)	67 (54.9 %)
Surgery Year		
<2000	38 (31.2 %)	38 (31.2 %)
≥2000 and <2010	49 (40.2 %)	60 (49.2 %)
≥2010	35 (28.7 %)	24 (19.7 %)
Number of Nodes Examined		
Median (Range)	15 (1, 52)	17 (1, 50)
IQR (1^st^ quartile, 3^rd^ quartile)	14 (9, 23)	15 (10, 25)
Tumor Location		
Ascending (Ascending, Cecum, Hepatic flexure, Transverse)	71 (58.2 %)	72 (59.0 %)
Descending (Descending, Sigmoid, Splenic flexure)	45 (36.9 %)	46 (37.7 %)
Colon NOS	6 (4.9 %)	4 (3.3 %)
T Stage		
T3	107 (87.7 %)	109 (89.3 %)
T4	15 (12.3 %)	13 (10.7 %)
MMR Status		
Deficient	11 (9.0 %)	21 (17.2 %)
Proficient	111 (91.0 %)	101 (82.8 %)
Lympho-vascular Invasion		
Yes	7 (5.7 %)	6 (4.9 %)
No	95 (77.9 %)	97 (79.5 %)
Not Reported	20 (16.4 %)	19 (15.6 %)

**Fig. 2:**
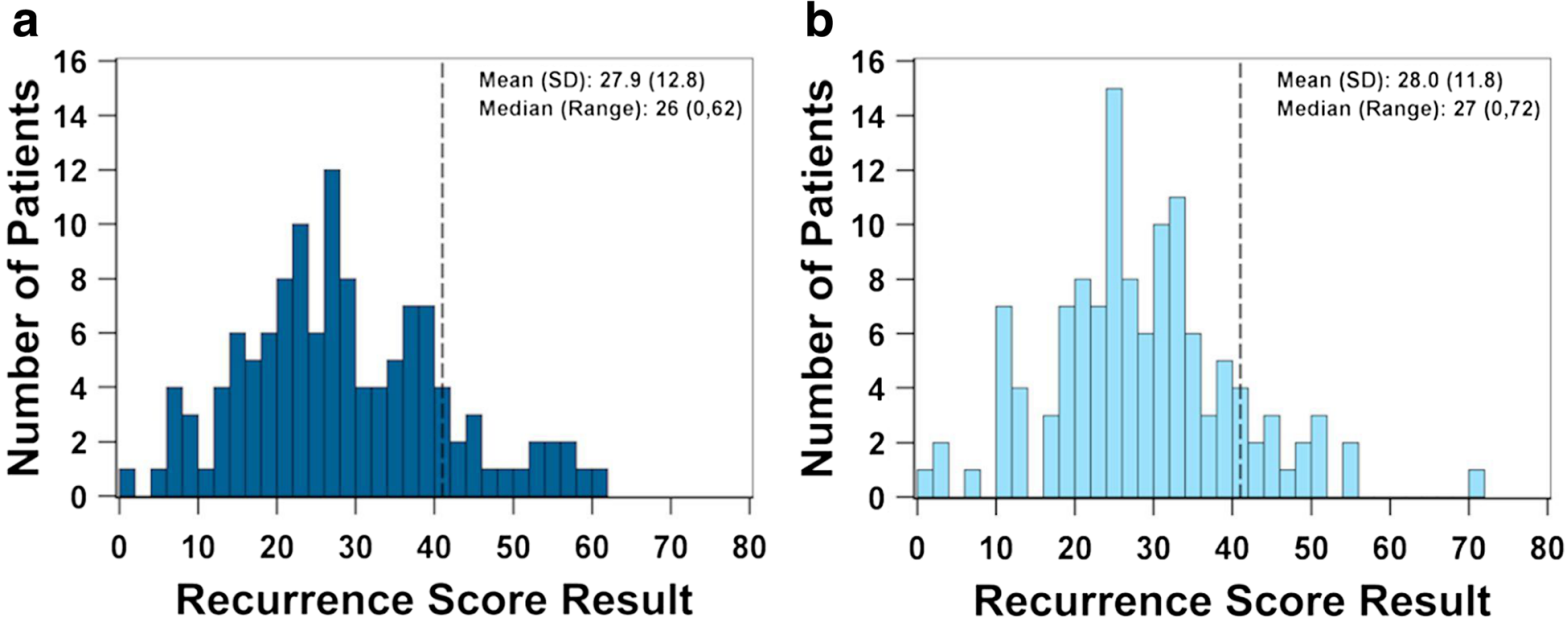
The distribution of Recurrence Score results for African-American patients and Caucasian patients. The number of patients with a given Recurrence Score result is displayed on the vertical axis; the Recurrence Score values are displayed on the horizontal axis. The mean, SD, median, and range of the Recurrence Score result are inset in each graph. Panel **a**: display of the Recurrence Score results for AA patients (*n* = 122). Panel **b**: display of the Recurrence Score results for CA patients (*n* = 122). The mean Recurrence Score result for African-American patients (27.9 ± 12.8) was not statistically different from that of Caucasian patients (28.1 ± 11.8). The vertical line at 41 represents the cutoff for the high Recurrence Score risk group [6]. SD: standard deviation; AA: African-American; CA: Caucasian

**Fig. 3:**
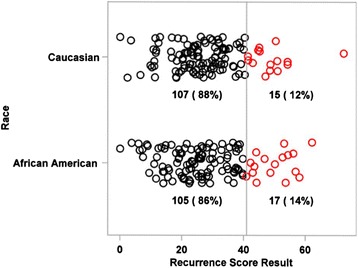
Recurrence Score result display by high recurrence risk cut off. The Recurrence Score result for each patient is displayed as a circle by ethnicity group. The Recurrence Score results >41 are colored red. The proportion of patients with high Recurrence Score values (≥41) was similar between the patient groups (17/122 African-American patients; 15/122 Caucasian patients)

**Fig. 4:**
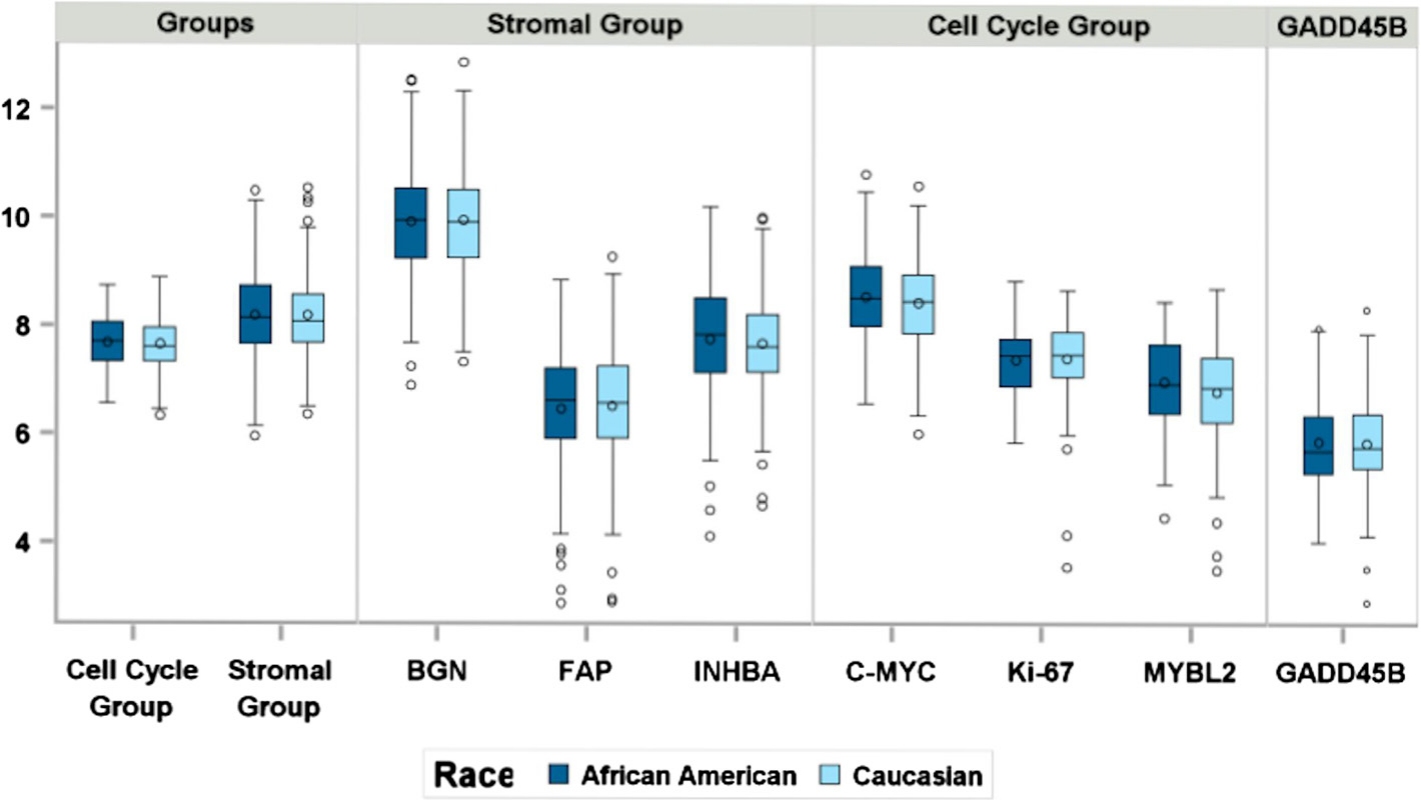
Distribution of the Gene Expression Levels for Gene Groups and Individual Genes. Gene expression variables, either single genes or gene groups, (Cell Cycle group, Stromal group, BGN, FAP, INHBA, Ki67, MYBL2, C-MYC, and GADD45B) were not significantly different between the racial groups

The mean Recurrence Score result was 27.9 ± 12.8 for the AA group and 28.0 ± 11.8 for the CA group and was not significantly different between groups (*p* = 0.93) (Fig. 2). The proportion of patients with a high Recurrence Score result (≥41) was similar between the groups: 17/122 (14 %) for AA patients and 15/122 (12 %) for CA patients (Fig. 3). None of the gene expression variables, either single genes or gene groups (Cell Cycle group, Stromal group, BGN, FAP, INHBA, Ki67, MYBL2, C-MYC, and GADD45B) was significantly different between the racial groups (*p* > 0.05 for all individual genes and gene groups) (Fig. 4).

Linear regression modeling of Recurrence Score result, gene groups, and individual genes with the explanatory variables including race, clinical covariates, and pathological covariates (including number of nodes examined, pathologic T stage, tumor grade, MMR status and LVI status) revealed no statistically significant association with patient race (data not shown).

## Discussion

Colorectal cancer is the third most common cancer in men and women in the United States and has the third highest mortality rate in both genders [1]. Relative to CA patients, AA patients with colorectal cancer have a higher incidence, higher mortality, and worse stage-specific outcomes [1, 9]. The reasons for these disparities are not well understood. The recent improvement in survival of patients with colorectal cancer across all stages has not been seen equally in AA and CA patients: the overall mortality rate has steadily abated in the last three decades, but the decline is less pronounced among AA compared to CA populations [1, 4]. The mortality rate among AA populations with colorectal cancer at all stages is in dispute, with some studies showing worse rates among AA patients with all stages, including early stage disease [10, 11] while others show the trend only for those with advanced stage disease [4, 9].

The higher colorectal cancer incidence and mortality rates among AA have been attributed to differences in socioeconomic status leading to lack of access to healthcare, although this has been disputed by others [2, 12]. Lack of access to healthcare, resulting in lower rates of screening and more advanced stage at presentation and consequently higher mortality, has been noted for the AA population [12, 13]. In Medicare beneficiaries, there is evidence for a difference in the treatment received for colon cancer [13]; for younger populations, there is also evidence of lower utilization of available treatment with chemotherapy and radiation among AAs [2, 14]. In contrast, studies of the Veterans Affairs Health Care Systems did not find a statistical difference in overall survival between AA and CA patients with colorectal carcinoma and suggested that uniform treatment of their patient population may be the reason for the lack of a difference in survival [15, 16]. Albain and colleagues reported that in prospective SWOG studies there was no difference in outcomes between AA and CA patients with respect to colon cancer, although AA patients with breast, prostate, or ovarian cancers had worse overall survival than CA patients; however, the small sample size and restriction of analysis to only those who were receiving adjuvant chemotherapy may have played a role in the differential results for colon cancer versus other tumor types [17].

Objective clinical factors have also been examined to identify underlying reasons for the racial disparity. A retrospective analysis of patients with colon cancer not receiving chemotherapy showed a higher mortality for AA subjects [10]. The difference in disease progression and worse mortality may be attributed to variations in tumor pathobiology in patients of different race and ethnicity [18, 19]. Several studies [5, 11], including a study conducted by University of Alabama, Birmingham investigators found that, although there was no difference in the distribution of tumor grade between AA and CA colon cancer patients, AAs with high-grade (poorly differentiated) tumors were at three times higher risk of dying of colorectal cancer as CA patients even after controlling for treatment and prognostic factors [20].

This is the first study to compare the 12-gene assay in colon cancer among AA and CA patients. This assay has been validated to predict the recurrence rate in patients with resected stage II colon cancer, whether treated with surgery alone or with surgery followed by adjuvant chemotherapy. We compared the Recurrence Score results of AA and CA patients who otherwise showed no differences in demographic or clinical factors. Although the number of genes assessed by the Oncotype DX assay is limited in number, the genes are known to be involved in cancer biology, therefore, the assay represents a useful, although not comprehensive, probe into the biology of these tumors. Among both AA and CA patients, there was a similar distribution of Recurrence Score results and equal numbers of subjects in both groups had high Recurrence Score results ≥41. The year of diagnosis was matched for the two groups to balance for any inequality in the number of lymph nodes harvested due to the introduction of new standards [21]. We did not exclude patients with fewer than 12 nodes examined because the aim of the study was to evaluate the expression of the genes underlying the biology of colon tumors in AA and CA patients, and not to evaluate the risk of recurrence. None of the gene expression variables, either gene groups or single genes (cell cycle group, stromal group, BGN1, FAP, INHBA1, Ki67, MYBL2, cMYC, and GADD45B), was significantly different between the patient groups.

A limitation of this study is that the assessment of genes was limited to those represented by the 12-gene assay. Accordingly, we cannot rule out the possibility of other underlying differences in tumor biology and molecular profiles between AA and CA patients. Further, as we did not have access to long-term clinical outcomes for patients in this study, we were unable to assess whether similarities in gene expression profiles for AA and CA patients are associated with similar clinical outcomes. Finally, although the AA and CA cohorts were matched based on year of diagnosis, age, and sex, the retrospective nature of this study does not preclude other potential sources of selection bias.

## Conclusion

The distribution of Recurrence Score results, gene expression levels of gene groups and individual genes were not significantly different between AA and CA patients; suggesting tumor biology, as measured by the 12-gene assay, did not differ between patient groups.

## Abbreviations

AA: African American
CA: Caucasian
MMR: Mismatch Repair
FFPE: Formalin-fixed Paraffin Embedded
LVI: Lymphatic Vascular Invasion
SWOG: Southwest Oncology Group
UAB: University of Alabama
UAMS: University of Arkansas for Medical Sciences
